# Diagnostic Reference Levels for Common X-ray Procedures in Peru

**DOI:** 10.7759/cureus.18566

**Published:** 2021-10-07

**Authors:** Andres Portocarrero Bonifaz, Caterina S Camarena Rodriguez, Ricardo Palma Esparza

**Affiliations:** 1 Radiation Oncology, University of Louisville, Louisville, USA; 2 Physics, Pontificia Universidad Catolica del Peru, Lima, PER; 3 Control de Calidad en Radiodiagnostico, QC Dose S.A.C, Lima, PER

**Keywords:** peru, quality control, dose, intraoral x-rays, general x-rays, drl, diagnostic reference levels

## Abstract

Diagnostic reference levels (DRLs) for X-ray procedures have been established in many countries since 1996. In Peru, data from the literature are used as guidelines as DRLs research is limited. The objective of this study is to analyze the parameters and variables which are used in radiological techniques such as kV, mAs, and type of machine (conventional or digital); study the geographical distribution of radiological X-ray machines, and establish DRLs in Peru. Two radiological procedures were considered, general X-rays (fixed and mobile) and intraoral X-rays (fixed, mobile, and portable). An Unfors RaySafe Xi detector (Unfors RaySafe AB, Billdal, Sweden) was used; air kerma was measured at a source to image distance that varied depending on the procedure, and the entrance skin dose was subsequently calculated using the Arcal XLIX formalism. The data were collected over a period of three years (2015-2017). Only results from the last evaluation during this period were taken into consideration for each X-ray machine. DRLs were calculated at 0.21 mSv, and 0.25 mSv for posterior-anterior chest examinations in conventional and digital machines, respectively; 4.39 mSv and 6.01 mSv for conventional and digital antero-posterior lumbar spine examinations, respectively; and at 4.21 mSv for the dental intraoral procedure. The largest amount of X-ray machines is concentrated in the city of Lima. These results reflect the standard of practice in Peru.

## Introduction

The absorbed dose, in matters of ionizing radiation, is the energy deposited per kilogram and it is represented by the Gray (Gy) in the international system of units. When the absorbed dose is multiplied by a weighting factor that depends on the type of radiation, it is known as the equivalent dose and its unit is the Sievert (Sv). For the case of X-rays, the weighting factor is one [1,2].

Research has shown that the difference between the minimum dose in one diagnostic center and the maximum dose in another can be up to 50 times as much [3]. Taking into account the principle of optimization, and having found these inconsistencies, the term "Diagnostic Reference Value" was proposed in 1990 [4].

The diagnostic reference level (DRL) value was first introduced in publication 73 of the International Commission on Radiological Protection (ICRP) [5]. The main objective was to establish a standard for the various techniques in radiological procedures [6]. In Peru, law 28028 [7], with its technical standard IR 003-2013, stipulates that the representative doses for patients must be determined for different radiographic procedures and that X-ray examinations must comply with the principles of justification, and optimization [8]. However, research in Peru for the establishment of DRLs is limited [9].

A DRL value can be of three types: national, regional, or local. National reference levels are established at the 75% percentile of the median DRL values ​​of health facilities in a country. However, ICRP mentions in its report 135 that an even greater optimization can be made if the 50% percentile is used. If an institution falls below this benchmark, it is recommended to focus on improving its diagnostic image quality, rather than reducing radiation levels [10].

In Peru, the organization that is dedicated to the regulation and control of ionizing radiation sources is the “Oficina Técnica de Autoridad Nacional” (OTAN). X-ray machines quality control services guarantee that the equipment is working correctly; it must be done annually for general radiology machines and every three years for intraoral dental X-ray machines [8].

Currently, one of the most widely used medical imaging quality control protocols in Peru is Arcal XLIX. The accepted limit for entrance skin dose for intraoral dental machines is 7 mGy [11]; while for general X-rays is 0.4 mGy for posteroanterior chest (PA), and 10 mGy for anteroposterior lumbar spine (AP) [12].

The objective of this study was to obtain Peruvian DRLs for intraoral and general X-rays (chest PA and lumbar Spine AP), study the parameters and variables which are used in radiological techniques such as kV, mAs, and type of machines, and analyze the geographical distribution of X-ray machines in the country. This article was previously presented as a Poster at the 2019 International Conference on Medical Physics Scientific Meeting on September 8, 2019.

## Materials and methods

A descriptive cross-sectional study was carried out. To obtain the study database, the quality control reports prepared by QC Dose SAC’s Department of Quality Control for Medical Imaging performed to X-ray machines of various public and private health centers were used; the data collection period lasted three years (2015-2017). To maintain the anonymity of each health facility, a code was assigned to each report. The information from 397 general X-ray machines and 254 intraoral dental machines was used.

In order to calculate the DRLs for common general radiology techniques (PA Chest and AP Lumbar Spine) and dental - intraoral examinations [13,14], the entrance skin dose was calculated with the data obtained from an Unfors RaySafe Xi detector with the method recommended by the Arcal XLIX protocol (Equation 1) [12]. This instrument has an air kerma measurement uncertainty of 5%; it was calibrated annually in the United States and was used throughout the study period [15].

For the analysis of the radiation technique parameters, the X-ray technicians were asked to obtain a radiographic image using the parameters of a standard adult patient (18 years old or older). The health facilities where pediatric evaluations are carried out were not taken into account for this study, nor were those that exceeded the permissible limits according to the Arcal XLIX protocol.

The entrance skin dose was calculated using Equation 1:

$D_{s}=K_{\mathrm{air}}\times \left ( \frac{\mathrm{SID}}{\mathrm{SSD}} \right )^{2}\times \mathrm{BSF}$

Equation 1. $D_{s}$ is the entrance skin dose. $K_{\mathrm{air}}$ is the air kerma value obtained from the Unfors RaySafe Xi detector. $\left ( \frac{\mathrm{SID}}{\mathrm{SSD}} \right )^{2}$ is the inverse correction factor, SID is the source-image distance and SSD is the source-surface distance. Finally, BSF is the backscattering factor. The values referenced in the Arcal XLIX for the BSF (PA Chest and AP Lumbar Spine: 1.4; Dental - Intraoral: 1.2), and for the standard thickness for chest and lumbar spine (23 cm), were taken into consideration.

As suggested by international literature, the national DRLs were calculated at the 75% percentile (P75) of the median of the diagnostic reference levels of health facilities distributed nationwide [10,16-18]. P50 was also calculated in order to have a reference for optimization purposes.

Statistical analysis was performed using IBM SPSS Statistics Version 24.0 (IBM Corp. Armonk, NY, USA) [19]. Various descriptive statistics and measures of central tendency were found. In addition to the dose, the parameters of the radiological techniques (kV, mAs) were studied for each different examination.

X-ray machines were also grouped into two areas: Lima (capital of Peru) or all the other departments combined together, and type of machines: digital or conventional.

## Results

The obtained results are presented below.

Table 1 establishes the DRLs (P75) for PA Chest (Conventional and Digital), AP lumbar spine (Conventional and Digital), and intraoral radiographs. Additionally, the number of health facilities evaluated, the standard deviation, and the value of P50 are presented.

**Table 1:** P50 and P75 of the median doses in health facilities, for the different X-ray techniques.

	Chest (C)	Spine (C)	Chest (D)	Spine (D)	Intraoral
Health Centers Number	221	167	65	62	182
Std (mSv)	0.09	1.98	0.11	2.6	1.51
P50 (mSv)	0.14	2.91	0.15	4.29	2.87
P75 (mSv)	0.21	4.39	0.25	6.01	4.21

**Figure 1:**
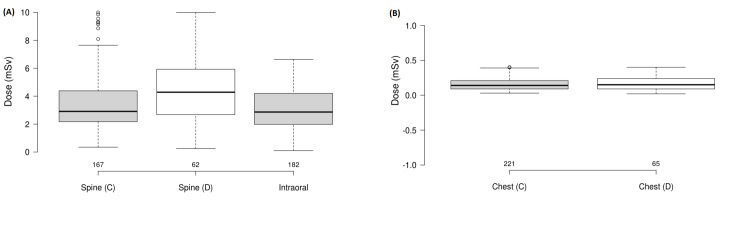
Box-plot graphs for the health centers' median dose values

Figure 1A shows the box-plot graphs for Conventional spine, Digital spine, and Intraoral X-ray examinations in order to better visualize the spread of the experimental data. Likewise, Figure 1B shows the box-plot graph for the Conventional chest and Digital chest.

**Figure 2:**
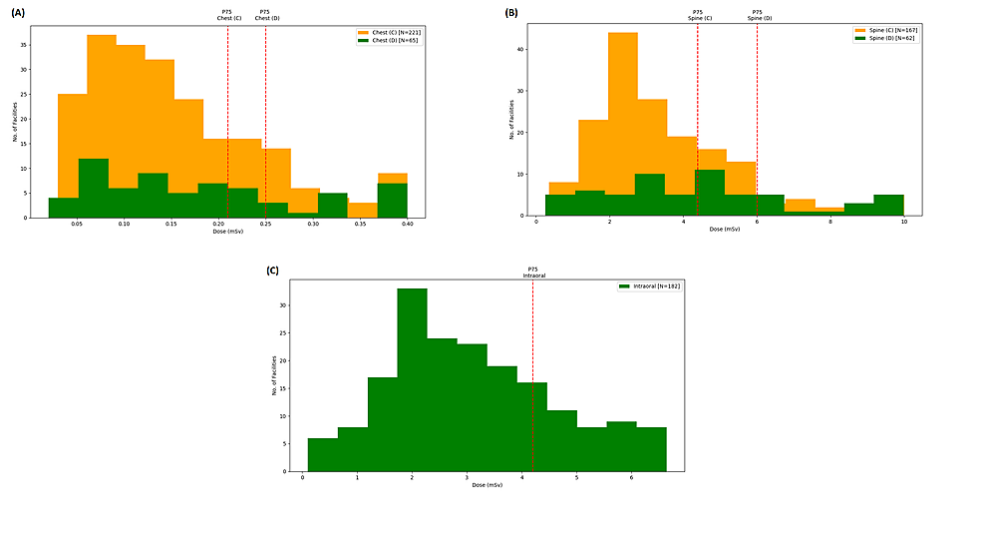
Frequency distribution of the health centers’ median doses

Figures 2A-2C show the frequency distribution of the median dose values for the health centers of the X-ray examinations considered for this study (PA Chest, AP lumbar spine, and intraoral). The value of the third percentile (P75) was marked in each histogram with a red dashed line according to the modalities shown in Table 1.

Table 2 contains the descriptive statistics for the parameters (kV and mAs) that are used in general X-ray for conventional and digital machines. Two types of examinations were studied in each of the cases, PA chest and AP lumbar spine.

**Table 2:** Analysis of general X-ray techniques for conventional and digital machines

	Chest (C)	Spine (C)	Chest (D)	Spine (D)
Min. (kV)	50	50	60	68
Max. (kV)	129	150	125	110
Mode (kV)	70	75	70	75
Std (kV)	16.4	9.28	17.86	8.01
Min. (mAs)	0.4	3.2	0.25	0.4
Max. (mAs)	40	123.75	16	80
Mode (mAs)	4	20	3.2	25
Std (mAs)	4.25	19.36	3.02	16.77

**Figure 3:**
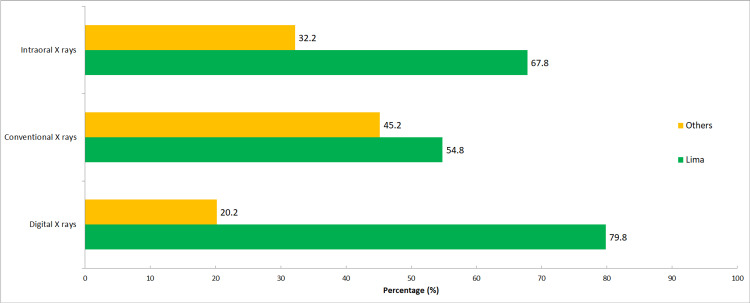
Geographical distribution of X-ray machines

Figure 3 shows the distribution of X-ray machines. It can be seen that the majority of digital machines is found in the capital (79.8%), as well as a greater number of intraoral (67.8%) and conventional (54.8%) machines, in comparison with all the other departments combined.

## Discussion

The DRLs for intraoral dental radiographs differ but stay within the same range from the results obtained by Kim et al. [17] in Korea, where the 75th percentile for an adult molar examination was 3.07 mSv. Likewise, the results obtained for general radiographic examinations vary from those obtained by Kiljunen et al. [18] in Slovenia, Sonawane et al. [20] in India, and Uniyal et al. [21] in India. The first authors obtained a value of the 75th percentile of lumbar spine AP of 7.98 mSv and of 0.35 mSv for chest PA. In the case of Sonawane et al. [20], they obtained values ​​of lumbar spine AP of 8.39 mSv and of 0.68 mSv for chest PA. Finally, Uniyal et al. [21] obtained values ​​of 8.55 mSv for lumbar spine AP and 0.43 mSv for chest PA. Moreover, DRLs also differ but were found on the same range when compared with Lithuania, whose reference diagnostic values ​​are 9 mSv for lumbar spine AP and 0.6 mSv for chest PA [22], Switzerland with values ​​of 8.7 mSv for lumbar spine AP and 0.2 mSv for chest PA [23], and the United Kingdom, whose DRLs are 5 mSv for lumbar spine AP and 0.15 mSv for chest PA [24]. It can be seen that the DRLs obtained in each country vary minimally. This is because the DRLs reflect local standards, and the optimization process must be approached from a national-level perspective [25,26].

Due to the differences in dose between conventional and digital general radiography X-ray examinations, which are characterized by their flexibility regarding dose and image quality [27], it is recommended to study both modalities separately and establish DRL for each modality. The DRLs for conventional and digital machines vary approximately in 2 mSv for lumbar spine AP (Table 1). It is important to emphasize that the ionizing radiation limits established in the protocols should not be exceeded, since when this occurs, the risk of generating a stochastic effect in the patient increases, and quality standards are not met.

On the other hand, when studying the parameters with which the various radiographic techniques are taken, discrepancies were found between health facilities (Table 2). It should be noted that the parameters depend on the X-ray machines and their condition; however, the modes of the various parameters give indicative measures in order to set a standard.

Likewise, it is worth mentioning that all departments combined have a greater national territory, as well as a greater amount of population, compared to the capital (Lima). However, most of the X-ray machines are found in the latter (Figure 3). Furthermore, 80% of the machines for digital radiography in Peru are in Lima. This may be due to the centralization that exists in Peru [28], and/or the lack of request for quality services for X-ray machines located outside the capital.

The greatest limitation of the present study is the size of the sample and the number of measurements taken for each machine; although the number of evaluated machines is high, it is recommended to increase the sample to obtain more reliable results, and to have more measurements in each machine; according to ICRP 135 [10], it is recommended to take measurements in at least 20 patients for general X-ray examinations. Finally, bias is introduced by the X-ray technicians when asked to irradiate based on a “Standard Adult Patient,” as they make an estimate of the technique based on experience.

## Conclusions

In conclusion, it is observed that DRLs for general X-rays and intraoral dental X-rays have been derived and can be used as references for optimization. With regard to radiological techniques, it was found that there are discrepancies between health establishments and that these parameters can impact the dose to the patient; for these reasons, continuous training is recommended in order to select the most appropriate technique. Finally, it is appreciated that there is a great technological concentration in the capital with respect to all the other departments, even more so when it comes to digital radiology machines.
